# Ophthalmia in Lying-In Hospitals

**Published:** 1923-04

**Authors:** G. C. Anderson

**Affiliations:** Deputy Medical Secretary. British Medical Association, 429 Strand, W.C.2


### Ophthalmia in Lying-in Hospitals.

SIR,—The section of Ophthalmology at the Newcastle-upon-Tyne Annual Meeting in July, 1921, passed a resolution to the following effect: "That, with a view to the prevention and adequate treatment of ophthalmia neonatorum occurring in lying-in hospitals, such hospitals should have upon the active staff an ophthalmic surgeon."

This resolution was referred to the Council for consideration and such action as might be deemed necessary. Believing that it might not always be possible for a particular lying-in hospital to have upon its active staff an opthalmic surgeon, the Council, upon the recommendation of the Hospitals Committee, passed the following resolution: "That, with a view to the prevention and adequate treatment of ophthalmia neonatorum occurring in lying-in hospitals, such hospitals should have available the services of an ophthalmic surgeon."

I have been instructed by the Council to forward this resolution to you.

G. C. ANDERSON,
Deputy Medical Secretary.

British Medical Association,
429 Strand, W.C.2.

